# The Role of Computed Tomography and Radiographs in the Management of Intertrochanteric Fractures

**DOI:** 10.5704/MOJ.2311.004

**Published:** 2023-11

**Authors:** VJ Patel, NB Patel, PM Tank, KA Upadhyay, KK Ashwin

**Affiliations:** 1 Department of Orthopaedics, Narendra Modi Medical College, Ahmedabad, India; 2 Department of Orthopaedics, Smt. NHL Municipal Medical College, Ahmedabad, India

**Keywords:** intertrochanteric fracture, radiograph, computed tomography, lateral wall

## Abstract

**Introduction:**

The Intertrochanteric fracture is a common hip trauma encountered in elderly patients. There is a lack of general agreement regarding its surgical management and choice of implant. Purpose of this study to conclude the final decision matrix regarding surgical management of intertrochanteric fractures based on parameters assessed on plain radiographs and CT scan.

**Materials and methods:**

We have retrospectively evaluated 55 patients with intertrochanteric fractures presented to our institute after informed consent with radiographs and CT scans between July 2017 to July 2018. Assessment of various parameters regarding fracture geometry and classification as well as measurement was done.

**Results:**

Mean lateral wall thickness in present study was 20.76mm. Incidence of coronal fragments was 90.9% and absence of coronal fragment in 5 patients. We noted the cases with anterior comminution had also a posterior comminution rendered the fracture unstable in almost 20 % cases.

**Conclusion:**

Better understanding of fracture geometry by combined used of radiograph and CT scan enhanced preoperative planning, choice of suitable implant, helps in reduction manoeuvre and improving quality of osteosynthesis.

## Introduction

Intertrochanteric Hip Fractures are commonly encountered in orthopaedics trauma. Incidences of these fractures are increasing with the advancing age due to osteoporosis and trivial fall.

For the individual patient these fractures may hamper day to day function and quality of life. About half of elderly patients may not regain pre-fracture level activity and hence independent living may not possible^1^. The mortality rate is higher in hip fractures and the overall one-year mortality is approximately 20-25% for the elderly patients with hip fractures^2^. Rising numbers of intertrochanteric fractures in patients with advanced age represent major health care burden to hospitals and health care providers as well as society.

Revision surgical intervention due to failure of index osteosynthesis is multi-factorial and it increased not only morbidity to patient but also health care expenditure. It also warrants the need of continuous future research for management of such fractures. Approximately 1.6 million hip fractures occur worldwide each year, by 2050 this number could reach between 4.5 million and 6.3 million^3^. In the current era operative intervention with suitable implants and accurate reduction with stable fixation which helps an individual to mobilise early and to minimise complications related to prolonged bed-rest and immobilisation; is the standard dictum of management. An efficient classification system for fractures helps in the decision making for the management. However, complications are sometimes also seen in the stable fracture variety classified alone on a plain radiograph. This could be a misunderstood fracture personality on a plain radiograph on many instances. When classification systems are unreliable, it is intricate to make choices in the management^4,5^.

Recent studies have highlighted the advantages of the availability of three-dimensional CT reconstructions for assessment of fractures^6,7^. The value of computed tomography (CT) for fracture classification has been studied for different types of complicated fracture patterns as conventional plain radiograph examination was considered to have limited accuracy in many instances. We evaluated radiographs and CT scans of 55 patients with intertrochanteric fractures admitted to our institute in order to confer the final decision matrix regarding surgical management of intertrochanteric fractures based on various parameters assessed.

## Materials and Methods

We retrospectively evaluated 55 patients with intertrochanteric fractures presented to our institute after informed consent with radiographs and CT scans between July 2017 to July 2018. All patients had been managed with hip fracture protocol norms of our institution. Out of 55 cases, 4 patients (7.2 %) had been undergone previous contra lateral trochanteric fixation. And 1 patient (1.8%) had past history of contra lateral malunited subtrochanteric fracture. A pre-operative CT scan of pelvis with both hips was obtained for all patients using 16 slice CT scanner machine [PHILIPS MX 16, Philips and Neusoft Medical Systems Co., Ltd, CHINA]. Measurement of different parameters were noted using diacom viewer of this system. We did not order post-operative CT scan.

**Fig 1:**
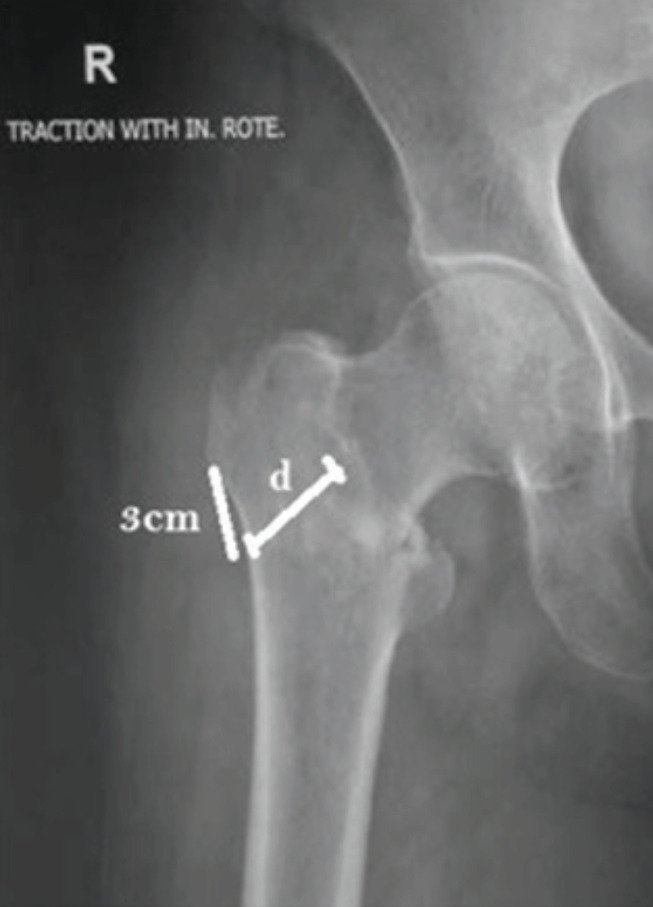
Showing the lateral wall thickness (d), defined as the distance in mm from a reference point 3cm below the innominate tubercle of the greater trochanter, angled at 135° upward to the fracture line (the midline between the two cortex lines) on anteroposterior projection.

**Fig 2:**
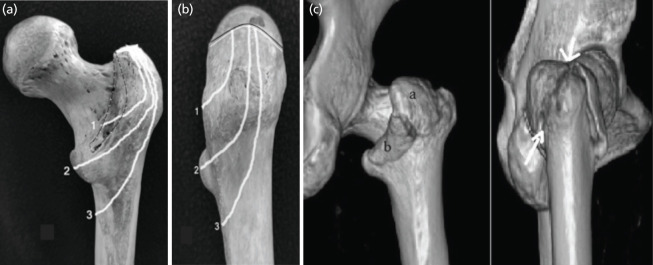
(a and b) Coronal fragment geometry where fracture line shows with white lines originating from trochanteric summit (black and white line in fig. b) exiting through ITC (1), LT (2) and PMC (3). (c) 3D CT scan NAKANO classification: type 1 3A fracture Coronal fragments: GT [a] LT [b] where its exit noted at ITC and LT (white arrow).

**Fig 3:**
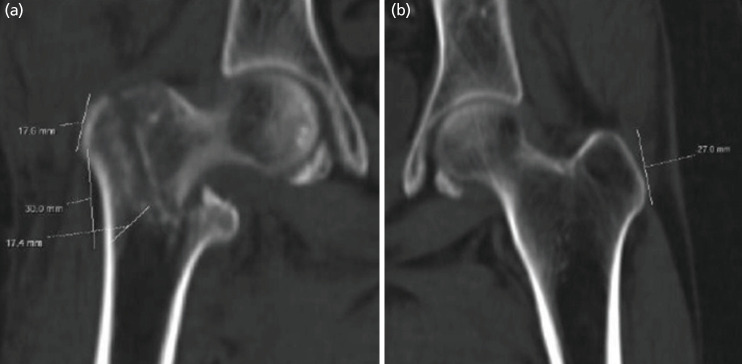
(a, b) AO Type A23 case: measurement via 2D CT scan where measured lateral wall thickness (17.4mm), ipsilateral greater trochanteric height 17.6mm, contralateral greater trochanteric height (27mm).

Our Inclusion Criteria were skeletally matured patients, pertrochanteric fractures with CT scan and radiograph evaluation and closed fractures. We excluded patients with neck femur fractures, past history of ipsilateral hip fracture or surgery, occult fractures which can be diagnosed only by MRI, open fractures and associated diaphyseal femur fractures. We assessed following parameters from the radiographs and CT scans; (i) AO Classification 2018: from radiograph and CT scan with 3D CT images^8^. (ii) Lateral Wall Thickness was measured from a reference point 3cm below the innominate tubercle of greater trochanter and angled at 1350 upward to the fracture line on a 2D CT scan images and radiographs^9^ (Fig. 1). (iii) NAKANO 3D CT Classification for Trochanteric Fractures^10^ (iv) Coronal Fragment Geometry: where entry and exit of fracture lines noted from 3D CT images^11^. (v) Height of the Greater Trochanter (Ipsilateral and Contralateral): The GT was defined as the extension of the lateral femoral surface proximal to the vastus ridge. We measured the height of intact GT extended from vastus ridge to the first fracture line crossing its lateral surface on 2D CT scan. (vi) Anterior Wall Comminution: noted from 3D CT scan images. (vii) Anteromedial Cortical Support Pattern: assessed from postoperative radiograph^12^. In recent 2018 AO COMPENDIUM, it separates the pertrochanteric fractures into two groups (A1 and A2) based on the amount of fragmentation and preserved thickness of lateral wall. A coronal fracture on the 3D CT scan was defined as a fracture line starting between the anterior and posterior margins along the trochanteric summit extending in the frontal plane on the lateral view. On the basis of 3D CT scan, coronal fragments are described based upon: The starting point and exit point of fractured fragment as well as the number of fragments. Based upon the exit points it divides into three elementary fragments namely GT (Greater Trochanter), GLT (Greater Trochanter, Lesser Trochanter), GLPC (Greater Trochanter, Lesser Trochanter, and Posteromedial Cortex). Further fracture of elementary fragments leads to multi fragmentary coronal fragments, and their nomenclature has been done according to exit levels of fracture lines. Diagrammatic representation of coronal fracture lines as observed on the lateral and posterior 3D CT reconstruction views (Fig. 2). The fracture line (white solid lines) starts at the trochanteric summit (TS) and exits either through ITC (1), LT (2), or PMC (3). The white and black double lines outline the TS as viewed from lateral. The black dotted line represents the intertrochanteric crest as viewed from posterior. On the console viewer of CT scan, three views (axial, coronal, and sagittal) were shown on the screen simultaneously. All measurements were taken on 2D CT images with corresponding sites (Fig. 3). Greater trochanter height measured bilaterally except in four cases where previous trochanteric fixation had been done where measurement had taken only on ipsilateral side. Such measurement had started from innominate tubercle/vastus ridge to the first fracture line exiting lateral aspect of greater trochanter on 2D CT coronal images.

**Fig 4:**
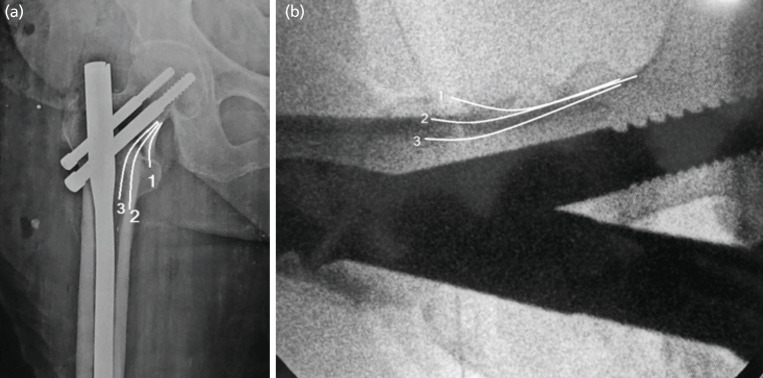
Shows anteromedial cortical support placements, (a) anteroposterior and (b) lateral view of radiographic images, respectively. Line 1 shows positive, line 2 shows neutral or anatomical and line 3 shows negative cortical reduction, respectively.

Anteromedial cortical support had been noted in three types of sub-groups^12^ (Fig. 4) those are (i) positive cortical support where proximal fractured fragment placed superomedially in context of upper medial edge of distal fragment. In lateral view, anterior cortices remained displaced anteriorly more than half of the cortical thickness or more than 2mm were considered as positive position. (ii) Neutral cortical support where proximal head-neck fragment placed in contacted smoothly with upper medial edge of distal fragment. In lateral view, anterior cortices remained smooth or displaced less than half of cortical thickness or less than 2mm was considered as neutral position. (iii) Negative cortical support where proximal fragment placed laterally to the upper medial edge of distal fragment. In lateral view, anterior cortices remained displaced posterior at more than half of the cortical thickness or more than 2mm was considered as negative position.

After evaluating all parameters, decision making for implant choice has been thoroughly cogitated as per patients’ profile individually in view of radiological parameter as well as associated medical co-morbidities. We would choose between extramedullary implants: DHS (dynamic hip screw) plate with additional CC screw (which act as a derotation screw) and intramedullary implants: Short Proximal Femoral Nail, Long Proximal Femoral Nail available at our institute. Patients had taken for surgery as earliest after anaesthetic clearance.

## Results

Analysis of 55 patients having intertrochanteric fractures assessed with plain radiographs and CT scans included in this study. In this study we evaluated AO classification from plain radiograph and CT scan along with 3D reconstruction. Overall distribution of classified fractures according to main three types described below where highest numbers of fractures coded into A2 variety followed by A3 and A1 (Table I). Combined used of plain radiographs and CT scan refined the fracture geometry and helpful for classifying the given fractures. Distribution according to AO subtypes described in table where highest numbers belonged to subtype A23 followed by A33 and A22. In our study 80% patients belonged to type 1 and 20 % patients belonged to type 2 Nakano 3D CT classification. Among type 1, 4 parts fracture comprised maximum patients. (40%) (Table II). The incidence of coronal fragments was 90.9% and absence of coronal fragment in 5 patients.

**Table I:** Distribution of fractures according to AO classification type

AO Fracture Type	31A1	31A2	31A3
Incidence	14.54% (8/55)	65.45% (36/55)	20.00% (11/55)

**Table II:** Distribution according to Nakano 3D CT classification

Sr No.	Nakano classification 3D CT	No. of Patient	% of Patient
1	TYPE 1 2B	2	3.64%
2	TYPE 1 3A	15	27.27%
3	TYPE 1 3B	3	5.45%
4	TYPE 1 3D	2	3.64%
5	TYPE 1 4 PART	22	40.00%
6	TYPE 2	11	20.00%
	Total	55	100.00%

We also noted that fragment involved greater and lesser trochanter both en-bloc in about four patients. Highest numbers of coronal fragment seen our study was GT fragment (15 cases) followed by GT and LT, GT and LPC fragments. Whereas highest number of coronal fragment exit seen at ITC level followed by ITC and PMC level (Table III). In present study Mean value of lateral wall thickness was 20.76mm. Out of 55 patients, there were fracture involving lateral wall in 11 cases where as lateral wall thickness >20.5mm were noted in 18 cases. Maximum numbers of patients had lateral wall thickness range from 15.6mm to 20.5mm, which is considered critical.

**Table III:** Shows incidence of different coronal fragments

Sr No.	Coronal Fragment	No. of Patient	% of Patient
1	GLPC	4	7.27%
2	GLT	3	5.45%
3	GLT+PMC	2	3.64%
4	GT	15	27.27%
5	GT+LPC	13	23.64%
6	GT+LT	13	23.64%
7	No fragment	5	9.09%
	Total	55	100.00%

We noted one patient had a thickness less than 10.5mm (Table IV). In this study we measured height of ipsilateral and contra lateral greater trochanter. Mean value of ipsilateral GT was 30.22mm and contra lateral GT was 51.69mm. we found cases with anterior comminution was about (21.81%) and a posterior comminution was about (70.90%). Four cases had no comminution. In A3 fractures anterior comminution was seen in 8 cases out of 12 cases (66.66%).

**Table IV:** Distribution of analysed data measuring lateral wall thickness

Sr no.	Measurements of lateral wall	No. of patients	percentage
1	5.6 – 10.5	1	1.81%
2	10.6 - 15.5	7	12.72 %
3	15.6 - 20.5	18	32.72 %
4	20.6 - 25.5	8	14.54 %
5	25.6 - 30.5	6	10.90 %
6	>30.5	4	7.27 %
7	Fractured Wall	11	20 %
	Total	55	

**Fig 5:**
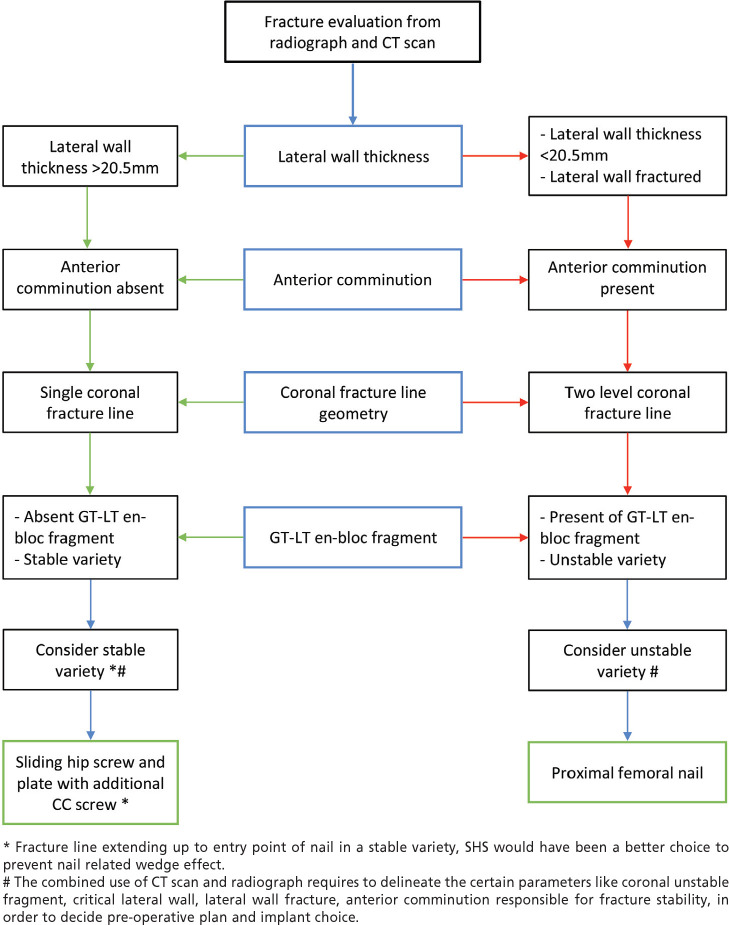
Fracture evaluation from radiograph and CT scan.

The cortical support pattern of reduction was analysed from post-operative reduction AP and lateral view radiographs where the highest numbers of pattern seen were the neutral cortical support followed by positive cortical support and the least numbers were seen with negative pattern. Lowest number of negative patterns in lateral view denotes no residual post-operative sagging of head and neck fragment (Table V). After considering above parameters in individual cases, we used long proximal femoral nail in 30 patients, short proximal femoral nail in 21 patients, dynamic hip screw devices in 4 patients (Fig. 5).

**Table V:** Described cortical support pattern distribution

Sr no	Cortical Support Pattern	No of patients		Percentage AP View	
		AP View	LAT View	AP View	LAT View
1	Positive	12	7	21.81%	25.45%
2	Negative	11	14	20%	12.72%
3	Neutral	32	34	58.18%	61.81%
	Total		55 Cases		

## Discussion

In this modern era of orthopaedics, rising number of Intertrochanteric Fractures are one of the important public health issues that warrant managing these fractures optimally on a very first operative attempt to prevent future risk of revision surgery where both morbidity and mortality would be a higher side with increased cost of intervention and inferior functional outcomes. Henceforth, to delineate the fracture personality thoroughly and selection of appropriate implant for osteosynthesis is of paramount importance.

Failure of the reconstructed intertrochanteric fracture depends on many variables like fracture type, quality of reduction, stability of fixation, degree of osteoporosis, compliance of patients and associated co-morbidities as well. For fixation of intertrochanteric fracture, it is extremely important to assess fracture pattern and its geometry in thorough depth. In many instances radiograph alone does not provide detailed information about the fractures. Additional use of CT scan provides detailed personality of fracture, degree of comminution and true measurements of various parameters, etc.

Recent AO compendium 2018 has been edited for intertrochanteric fractures with inclusion of additional variable of lateral wall thickness. Along with level of fragmentation, Lateral Wall thickness less than 20.5mm includes A2 fractures while more than 20.5mm includes A1. Fracture were classified as per AO compendium 2018 with help of both radiograph and CT scans. Differences in different subtypes as compared to other studies may be due to previous older coding of AO classification by other studies where lateral wall thickness was not included and involvement of younger population with high energy of trauma. While using dynamic hip screw, Gotfried and Palm et al both emphasised importance of integral lateral wall^13,14^. Hsu *et al*^9^ described lateral wall thickness less than 20.5mm should not be treated alone with sliding hip screw alone. Tan *et al*^15^ concluded that superolateral support is one of the most important factors than the medial calcar buttress. They also suggested that CT scan is important in pre-operative planning.

Definition of lateral wall thickness is somewhat controversial, and its measurement doesn’t hold true even in all radiographs especially the degree of rotation gives different value of lateral wall. Measurement of such lateral wall thickness has to be taken on 2D CT axial images would be advisable and appropriate than on plain radiographs. Fixation failure and rate of revision in intertrochanteric fractures attributed to intactness of lateral femoral wall. It is an important indicator biomechanically in order to decide further management plan.

Another important predictor of success could be the presence of the coronal fragments. Cho JW *et al*^11^ first described definition of Coronal fragments of intertrochanteric fractures and its incidence in their 3D CT scan-based study. In our study we also noted that it was difficult to interpret coronal fragment from radiographs alone. The 3D CT scan evaluates coronal fragment geometry meticulously (Table VI). Shoda *et al*^16^ noted that radiograph alone fetched obscured information about fracture whereas CT scan defines it entirely and modifies 3D CT classification by Nakano.

**Table VI:** Comparison of incidence of coronal fragments with other study

Sr No.	Coronal Fragment	Cho JW et al11	Present study
1	GLPC	20.28%	7.27%
2	GLT	13.76%	5.45%
3	GLT+PMC	4.34%	3.64%
4	GT	25.36%	27.27%
5	GT+LPC	22.46%	23.64%
6	GT+LT	13.76%	23.64%
	Total incidence of coronal fragment	88.4% (138/156)	90.9% (50/55)

Incomplete information of coronal fragment may lead to additional use of imaging intra-operatively, wrong choice of implant, increased length of procedure and inconsistent implant trajectory which ultimately hampers the quality of reduction and quality of osteosynthesis. In case of displaced coronal split fragment of greater trochanter, we used a femoral shaft as a reference entry point instead of tip of greater trochanter. If using a tip of GT as a reference point, free fragment usually misguides the entry of nail trajectory leading to varus reduction and subsequent failure of osteosynthesis. Moreover, we noted that coronal fragment involving fractured GT in almost 90% of cases. Loss in height of greater trochanter increased chances of compromised lateral wall, comminution of greater trochanter and reduced the stability of fractures. Height of greater trochanter is also known as lateral wall height^17^. We also noted that height of the GT is reduced when fracture line become more horizontal leaving very short amount of GT which would be act as a superolateral buttress. This is one of the key contributing factors in mechanical failure which should be considered while choosing extramedullary vs intramedullary implants.

Chang *et al*^12^ showed the anteromedial cortical support reduction is a functional non-anatomic buttress reduction which confers secondary stability. Positive/positive and positive/neutral cortical patterns are reliable definitive support where as negative cortical pattern on lateral view is highly predictable for post-operative loss of anteromedial cortical support leading to excessive sliding of dynamic hip screw and failure of osteosynthesis. Higher differences in cortical support pattern in neutral cortical support might be due to anatomical reduction attainment in our study. We noted that lower rate of negative cortical support in lateral view due to optimal reduction intra-operatively where no posterior sagging of head-neck achieved. Tsukada *et al*^18^ concluded that comminution anterior cortex associated with higher risk of cutting out when treated with Sliding Hip Screw (SHS).

**Fig 6:**
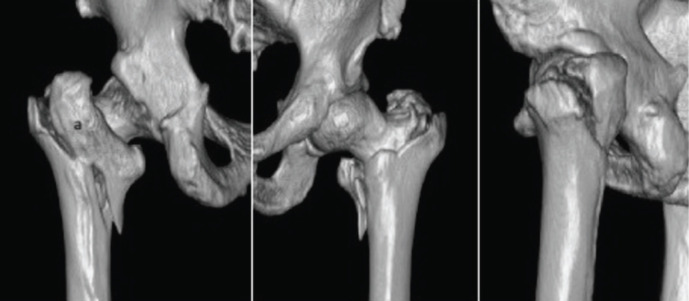
Coronal fragment: GLPC (a), where its exit noted at PMC (GT-LT En-bloc fragment).

In our study we also noted that whenever the comminution of anterior cortex was present, there was presence of posterior comminution too. Restoration of cortical continuity is limited by comminution and fragmentation of anterior wall. The higher incidence of anterior comminution had been seen in A3 fractures. Hence these varieties consider as a highly unstable pattern and superiority of intramedullary fixation to treat them reviewed by many recent literatures. Moreover, we noted third fracture fragments involved greater and lesser trochanter en-bloc in four patients. Nakano pointed out in their study that this large fragment renders the fracture unstable. This large GT-LT en-bloc fragment tends to be misdiagnosed on plain radiographs (Incidence in present study ~7%, (Fig. 6).

Choice of implant in intertrochanteric fractures has been controversial debate even in recent literature it shows inconsistency in different modalities with differences in choices of implant. Most of the recent studies suggested use of SHS devices in stable fracture pattern and considered as gold standard. In unstable variety of intertrochanteric fractures use of intramedullary devices confers superior mechanical construct with maximum preservation of fracture biology. Stable construct in such fractures prevent excessive medialisation of shaft and varus collapse and aid in maintaining of good reduction which ultimately offers early mobilisation. Decision regarding using of longer nails depends upon the extension of fracture line to subtrochanteric region, bowing of femur, highly unstable variety. Shen *et al*^19^ assessed the influence of a pre-operative CT study that resulted in shorter operating times for im nailing for hip fractures.

Quality of reduction can be surgeon related variable which can be improved by better understanding of radiographs and CT scans. Quality of reduction remains of paramount importance regardless of implant choice. Inferior quality of reduction restricts patient from early mobilisation and rehabilitation protocol leading to secondary complications, and also hampers the quality of fixation and chances of failure leading to revision surgery. Our study is a not a comparative study as direct comparison to other studies was not done as well as Relatively smaller number of patients and quantitative analysis of osteoporosis was not studied. Clinical outcome of analysed patients on a larger scale needs to be verified for future inclusion of imaging modalities.

## Conclusion

Decision making in the treatment of intertrochanteric fractures requires better understanding of fracture geometry, proper pre-operative planning, surgical expertise, familiarity with implants and associated co morbidities. Plain radiograph does not always provide requisite lateral wall details, fracture patterns and coronal fragment geometry. When CT scan used pre-operatively, it provides essential fracture assessment along with coronal fracture lines, coronal unstable fragment, precise lateral wall thickness, anterior wall comminution, occult fracture lines that exit the entry site for intramedullary nail. Information provided by CT scan is superior and crucial in guiding appropriate pre-operative planning and choice of suitable implant for a particular patient.
